# ST Segment Elevation Myocardial Infarction in Coronary Arteries with Massive Ectasy

**DOI:** 10.5935/abc.20160093

**Published:** 2016-09

**Authors:** Ana Rita G. Francisco, José Duarte, Miguel Nobre Menezes, José Marques da Costa, Pedro Canas da Silva, Fausto J. Pinto

**Affiliations:** Centro Hospitalar Lisboa Norte, Hospital de Santa Maria, Lisboa - Portugal

**Keywords:** Acute Coronary Syndrome, Thrombectomy, Coronary Artery Disease, Coronary Aneurysm

**Figure 1:**
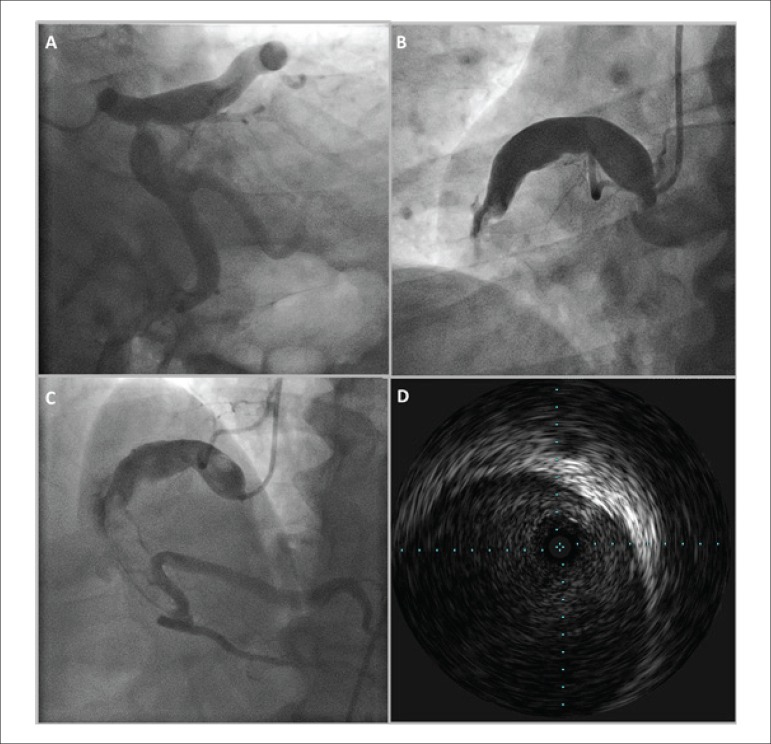

A 69-year-old caucasian male with a history of hypertension, dyslipidemia, obesity and tobacco use was admitted due to an inferior ST-segment elevation myocardial infarction with two hours evolution. He was treated with aspirin, clopidogrel and unfractioned heparin, and an emergent transradial coronary angiography was performed. Ectasic dilatation of left main, left anterior descending and circumflex arteries were documented, with distal TIMI 2 flow (Figure 1A). The dominant right coronary artery (RCA) was massively dilated proximally and occluded in the mid segment (Figure 1B).

Percutaneous coronary intervention of RCA was attempted, using an AL 1 6 Fr catheter. Thrombus aspiration and balloon dilation of the mid/distal segments were performed, with distal flow recovery (TIMI 2) (Figure 1C). Given the massive ectasy, no stent was implanted. After five days of triple therapy (aspirin, clopidogrel and warfarin) a new coronariography was performed: intracoronary echocardiography revealed an ectasic RCA, with recanalyzed thrombus. The maximum diameter was 14 mm proximally and 8 mm in the middle segment, at the previous occlusion site (Figure 1D).

The patient was managed conservatively with long-term anticoagulation.

Giant coronary artery aneurysms (CAA) are rare and convey a risk of acute coronary syndromes, usually due to local thrombosis. In addition to antiplatelet therapy, anticoagulation is recommended, with surgical or percutaneous excision of CAA in patients with ischemia or a significant change in dimension over time. In this case, given the diffuse character of these lesions, this approach was unsuitable. In recurrent cases, the use of peripheral, self-expanding stents, may be considered.

**Vídeo:** Watch the videos here: http://www.arquivosonline.com.br/2016/english/10703/video_ing.asp

